# Mechanically Robust and Repairable Superhydrophobic Zinc Coating via a Fast and Facile Method for Corrosion Resisting

**DOI:** 10.3390/ma12111779

**Published:** 2019-05-31

**Authors:** Junfei Ou, Wenhui Zhu, Chan Xie, Mingshan Xue

**Affiliations:** School of Materials Science and Engineering, Nanchang Hangkong University, Nanchang 330063, China; 70299@nchu.edu.cn (W.Z.); 70362@nchu.edu.cn (C.X.)

**Keywords:** superhydrophobic, zinc-rich coating, cold galvanized coating, durability

## Abstract

Zinc coatings and superhydrophobic surfaces have their own characteristics in terms of metal corrosion resistance. Herein, we have prepared a robust and repairable superhydrophobic zinc coating (SZC) based on a widely commercially available cold galvanized paint via a fast (within 10 min) and facile process for corrosion resistance. Specifically, the cold galvanized paint was sprayed onto the iron substrate, followed by acetic acid (HAc) etching and stearic acid (STA) hydrophobizing. The as-obtained sample was coded as Fe-Zn-HAc-STA and possessed an apparent contact angle of 168.4 ± 1.5° as well as a sliding angle of 3.5 ± 1.2°. The Fe-Zn-HAc-STA sample was mechanically durable and easily repairable. After being ultrasonicated in ethanol for 100 min, the superhydrophobicity was still retained. The Fe-Zn-HAc-STA sample lost its superhydrophobicity after being abraded against sandpaper with a load of 100 g and regained its superhydrophobicity after HAc etching and subsequent STA hydrophobizing. The corrosion resistance of the SZC was investigated by immersing the Fe-Zn-HAc-STA sample into the static or dynamic aqueous solution of NaCl (3.5 wt.%) and the lasting life of the entrapped underwater air layer (EUAL) was roughly determined by the turning point at the variation curve of surface wettability against immersion time. The lasting life of the EUAL iwas 8 to 10 days for the SZC in the static NaCl solution and it decreased sharply to 12 h in a dynamic one with the flow rate of 2 and 4 m/s. This suggests that the superhydrophobic surface provided extra corrosion protection of 8 to 10 days or 12 h to the zinc coating. We hope that the SZC may find its practical application due to the facile and fast fabrication procedure, the good mechanical durability, the easy repairability, and the good corrosion protection.

## 1. Introduction

Iron and steel are the most common metals used in industry. However, the corrosion and damage of iron are serious; therefore, the anti-corrosion is an important issue in the iron industry [1]. People are using conversion coatings, such as chromate conversion coating and phosphate conversion coating, to improve its corrosion resistance. These conversion coatings have found wide practical application, however, also possess certain inherent disadvantages such as the carcinogenicity caused by the hexavalent chromium [2] and the high energy costs caused by the high treating temperature [3].

The cold zinc-spraying coating is an environment-friendly alternative with the standard electrode potential of −0.76 V, which is more active than iron (−0.44 V) [4]. When the coating is attacked, the zinc powder is corroded as the anode first, and the base iron is protected as the cathode, so the corrosion rate of iron can be slowed down significantly.

Superhydrophobic surface (SHS) with an apparent contact angle above 150° and a sliding angle below 10° is another newly-developed corrosion resistance strategy [5,6,7,8,9,10,11,12,13,14,15]. Once the superhydrophobic sample is immersed into water, a layer of air will be entrapped at the solid/liquid interface, which hinders the penetration of corrosive species (such as the Cl^−^ ions dissolved in water) to reach the metallic substrate and improve the corrosion resistance greatly [5].

To combine the sacrificing anode effect of zinc coating with the air barrier effect of SHS, Zhang et al. [16] and Brassard et al. [17] have fabricated superhydrophobic zinc coatings (SZC) via electrochemical zinc deposition and subsequent surface passivation by polypropylene or silicone polymer. Such SZC samples improve the anti-corrosion performance of iron or steel significantly. However, the zinc deposits are obtained via an electrochemical deposition, which makes the fabrication of the SZC sample instrument-dependent.

Herein, we aimed to fabricate an SZC sample via a more facile method. The metallic zinc layer was obtained by spraying the cold galvanized paint onto the iron substrate, followed by acetic acid etching and subsequent stearic acid hydrophobizing. The procedure was time-saving (within 10 min) and easy to perform. The mechanical durability was assessed by ultrasonication in ethanol and abrasion against sandpaper. The corrosion resistance was investigated by immersing the Fe-Zn-HAc-STA sample into the static or dynamic aqueous solution of NaCl (3.5 wt.%), and the variation in surface wettability, surface morphology, and surface composition was monitored.

## 2. Experimental

### 2.1. Sample Fabrication

The cold galvanized coating (Shanghai Roval Zinc-rich Coating Co, Ltd., Shanghai, China) was shaken evenly and sprayed directly onto a clean iron substrate (2.5 mm × 2.5 mm × 1 mm). Then, the sample was dried at room temperature for 12 h to obtain a uniform cold galvanized coating. The sample was immersed into the aqueous solution of acetic acid (HAc, 18 wt.%) followed by rinsing with ultrapure water and blown dry with nitrogen. Finally, the sample was immersed into the ethanol solution of stearic acid (STA, 0.01 M, chemically pure, Shantou Xilong Chemical Co. Ltd., Shantou, China) for 30 s and blown dry with hot air. To obtain the optimum superhydrophobicity, the immersion in STA was repeated twice. For convenience, the sample after spraying, etching, and hydrophobizing was coded as Fe-Zn, Fe-Zn-HAc, and Fe-Zn-HAc-STA, respectively.

### 2.2. Characterization

The surface morphology of the sample was characterized by field emission scanning electron microscopy (FE-SEM, Nova, Nano-SEM 450, FEI, Hillsboro, OR, USA; at 1 kV) with attached energy dispersive spectroscopy (EDX, FEI Quanta 200, USA; at 20 kV) under a vacuum environment. The chemical compositions and valence states of the samples were measured using X-ray photoelectron spectroscopy (XPS, Physical Electronics, Chanhassen, MN, USA, PHI-5702) with the Al Kα X-ray source (hν = 1486.6 eV) at a base pressure of 10^−7^ Pa. The surveyed (enlarged) spectra were recorded with a pass energy of 20 (5) eV and an energy step of 0.5 (0.05) eV. The binding energy of adventitious carbon (C1s: 284.8 eV) was used as a basic reference. The crystal orientation of the samples was characterized by X-ray diffraction (XRD, Bruker D8 ADVANCE, Karlsruhe, Germany) using CuKα radiation from 10 to 80° (2 theta) at 4 °/min. The powders scraped from the surface of the samples were recorded by Fourier Transform Infrared spectrometer (FTIR Nicolet IS10, Thermo Scientific, Waltham, MA, USA) by the KBr pellet methods with air as the reference. A resolution of 4 cm^−1^ and 32 scans were chosen. The surface wettability was measured by a contact angle meter (CAM, DSA 20, Krüss, Hamburg, Germany) with a computer-controlled liquid dispensing system and a motorized tilting stage at ambient temperature (20 °C). For apparent contact-angle measurements, a 10 μL water droplet was used with the Laplace-Young fitting. Advancing and receding angles were measured by the addition and subtraction of 5 μL of water to/from the 10 μL droplets sitting on the surfaces. For the sliding angle measurement, the water droplet (10 μL) was dripped onto the sample, which was then rotated until the water drop slid away. The critical angle where a water droplet began to slide down the inclined plate was measured to be the sliding angle. Each reported datum was an average of at least three measurements on the surface.

The adhesion of the coating to the iron substrate was assessed according to the standard of GB9286-98 by a crosscut tester (QFH-A, Dongguan Sanhe Instrument Manufacturing Factory, Dongguan, China). After the crosscut pattern at 90° was formed on the coating, the sample was brushed lightly with a soft brush to remove excess debris from the surface. A transparent tape was applied to the cut surface and rubbed with a rubber to ensure good contact with the coating and then removed after 90 s. Samples were evaluated under a magnifying glass (7×) and a bright light, and rated according to the American Society for Testing Materials (ASTM) rating scheme.

The variation of the surface wettability of the sample against ultrasonication (40 kHz, 100 W) in ethanol or against abrasion under a load of 100 g in contact with sandpaper (600 grits) was used to test the mechanical durability. The corrosion resistance was measured by immersing the sample into the static or dynamic aqueous solution of NaCl (3.5 wt.%) and the variation of surface morphology, chemical composition, and surface wettability was measured by FE-SEM, EDX, and CAM, respectively.

## 3. Results and Discussion

### 3.1. Wettability, Surface Morphology, and Surface Chemistry

**Figure 1:**
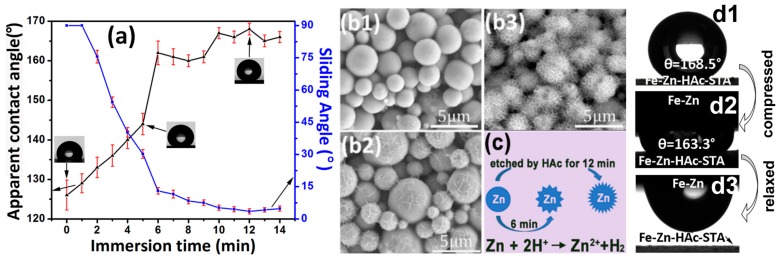
Variation of the surface wettability for the Fe-Zn-HAc-STA sample against immersion time in the aqueous solution of HAc (**a**). Scanning electron microscopic images of the Fe-Zn sample (**b1**), the Fe-Zn-HAc with an etching time of 6 min (**b2**) and 12 min (**b3**). Representative for zinc particles in images (**b1**–**b3**) and the etching reaction (**c**). Compression of a water droplet on the Fe-Zn-HAc-STA sample with the etching time of 6 min in HAc by a Fe-Zn sample (**d1**–**d3**).

The surface wettability of the Fe-Zn-HAc-STA sample varies against the etching time in the aqueous solution of HAc. The apparent contact angle or sliding angle levels off after rising or falling steeply for the first 6 min to a value of 161.8 ± 3.0° or 13.0 ± 1.5°, respectively (Figure 1a). As shown in Figure 1(b1), the so-sprayed cold galvanized paint was composed of smooth zinc micro-particles (MPs). After for 6 min, the MPs became rough with nano-wrinkles (Figure 1(b2)). As the etching time increased to 12 min, the nano-wrinkles became much denser (Figure 1(b3)) and the superhydrophobicity was optimum (apparent contact angle of 168.4 ± 1.5° and sliding angle of 3.5 ± 1.2°). The formation mechanism for these nano-wrinkles was due to the un-uniform etching between HAc and zinc as observed by Qian et al. [18]. It is explained that there are numerous dislocation defects in metals, which possess relatively higher energy and are prone to be attacked by chemical etchants such as HAc. Therefore, the etching rate in these dislocation sites was quicker and consequently, micro/nano-hierarchical surface structures were generated (Figure 1c).

The wetting state of the Fe-Zn-HAc-STA sample with an etching time in HAc for 6 min was further assessed by measuring the contact angle hysteresis (CAH), which was approximately 4° and equivalent to the difference between the advancing angle (approximately 169°) and the receding angle (approximately 165°). The Fe-Zn sample possessed an advancing angle of approximately 143° and a receding angle of about approximately 35°. A water droplet on the Fe-Zn-HAc-STA sample was compressed by the Fe-Zn sample and the typical images are shown in Figure 1d. After compression, the contact angle decreased slightly from 168.5° to 163.3°; after relaxation, the water droplet adhered to the upper surface and there was no residual on the lower superhydrophobic surface. These analyses suggest that the water droplet on the Fe-Zn-HAc-STA sample with an etching time in HAc of 6 min was in the Cassie state. The hierarchical surface roughness was helpful to resist the destabilization from Cassie wetting state to Wenzel state [15].

**Figure 2:**
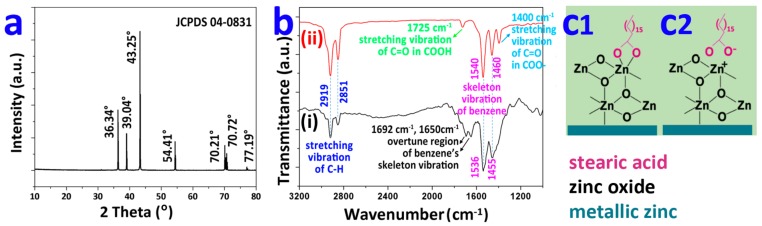
X-ray diffraction pattern of the Fe-Zn sample (**a**). Fourier transform infrared spectra of the Fe-Zn sample (curve i) and the Fe-Zn-HAc-STA sample (curve ii) (**b**). A possible mechanism for stearic acid anchored to the oxidized zinc: coordination (**c1**) or ionic (**c2**) bonding.

The XRD pattern (Figure 2a) for the Fe-Zn sample shows the strongest diffraction peak at 2θ of 43.25°, and weak ones at 36.34°, 39.04°, 54.41°, 70.21°, 70.72°, and 77.19°. These peaks are consistent with the standard PDF card of zinc (JCPDS 04-0831). Therefore, the main component of the Fe-Zn sample was zinc powder. Figure 2b shows the FTIR spectra of the Fe-Zn sample and the Fe-Zn-HAc-STA sample. The peaks at 1536 cm^−1^ and 1455 cm^−1^ in curve (i) for the Fe-Zn sample were attributed to the skeleton vibration of the benzene ring [19]. This was most likely attributed to the benzene ring in the bisphenol A epoxy resin, which was a routine ingredient for the cold galvanized paint to enhance the adhesion to the substrate. The peaks at 2919 cm^−1^ and 2851 cm^−1^ in curve (ii) were attributed to the C–H in epoxy resin [19], which became much stronger for the Fe-Zn-HAc-STA sample (curve ii). These stronger peaks were due to the anchored STA molecules abundant with C–H bonds. Compared with curve i for the Fe-Zn sample, two peaks emerged in curve ii for the Fe-Zn-HAc-STA sample, viz., a weak peak at 1752 cm^−1^ attributed to the carboxyl groups (COOH) [20] and a strong peak at 1400 cm^−1^ attributed to the deprotonated carboxyl groups (COO^−^) [17]. The stronger signal at 1400 cm^−1^ suggests that most of the carboxyl groups are deprotonated to facilitate the anchoring of STA to the Zn MPs via coordination bonding (Figure 2(c1)) or ion bonding (Figure 2(c2)) [21].

**Figure 3:**
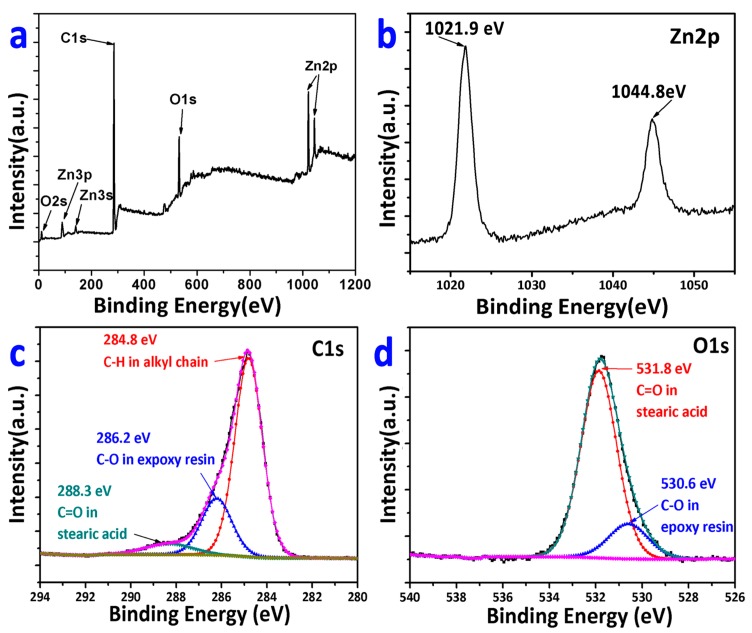
XP spectra for the Fe-Zn-HAc-STA sample: survey spectrum (**a**), Zn 2p spectrum (**b**), C 1s spectrum (**c**), and O 1s spectrum (**d**).

Figure 3 shows the XP spectra of the Fe-Zn-HAc-STA sample. In the survey spectrum (Figure 3a), peaks were attributed to the elements of oxygen, carbon, and zinc emerge. The Zn 2p peaks were located at 1021.9 eV and 1044.8 eV, which were higher than the binding energy of Zn (0) and ascribed to the Zn (II) species [22]. This suggests that, due to its high reactivity, the outermost surface of Zn MPs was oxidized. The as-formed oxide layer facilitated the anchoring of STA to the Fe-Zn-HAc sample just as shown in Figure 2c. The C 1s spectrum was deconvoluted to three components, viz., the C–H bonding at 284.8 eV, the C–O bonding at 286.2 eV, and the C=O bonding at 288.3 eV [23]. The deconvoluted peak at 531.8 eV for O 1s was due to the O=C group and the one at 530.6 eV was due to the O–C bond, both of which once again prove the presence of STA on the sample surface.

### 3.2. Mechanical Durability and Repairability

**Figure 4:**
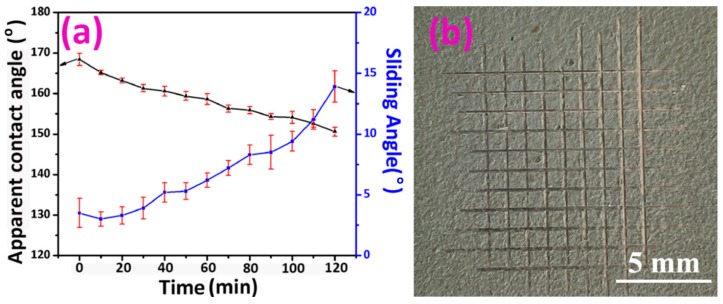
Variation of surface wettability for the Fe-Zn-HAc-STA sample against ultrasonication time in ethanol (**a**). The surface appearance after cross-cut testing (**b**).

Ultrasonication has been widely used to evaluate the mechanical durability of the superhydrophobic samples [24,25,26,27,28]. Bubble implosions by quick forming and violent collapsing generate shock waves and exert mechanical forces on the surface. Herein, the Fe-Zn-HAc-STA sample was immersed in ethanol, and the variation of surface wettability was plotted (Figure 4a). After 120 min, the apparent contact angle decreased slightly from 168.4 ± 1.5° to 150.6 ± 1.1° and the sliding angle increased from 3.5 ± 1.2° to 13.9 ± 1.3°. The most durable sample in the references was reported by Peng et al., which retained its superhydrophobicity after ultrasonication in ethanol for 14 h [21]. As reported by Liu et al. [25] and Huang et al. [26], the superhydrophobic samples can withstand ultrasonic treatment of 1 and 2 h, respectively. As reported by Liu et al. [27], after ultrasonication of 30 min, the apparent contact angle decreased from 167° to 141°. As reported by He et al. [28], after ultrasonication of 10 min, the apparent contact angle decreased from approximately 153° to approximately 136°. Comparing with these References [25,26,27,28], we can say that the Fe-Zn-HAc-STA sample was mechanically robust.

The good mechanical durability was due to the strong interlayer interaction. To evaluate its adhesion strength to the iron substrate, the Fe-Zn-HAc-STA sample was crosscut and peeled off by Scotch tape according to the standardized coating adhesion test method (ASTM D 3359) [29]. As shown in Figure 4b, the edges of the cuts were completely smooth and none of the squares of the lattice were detached. Therefore, the Zn-HAc-STA coating adhered to the iron substrate in the level of 5B. The high adhesion strength was due to the epoxy resin, which was a routine ingredient in cold galvanized paint as confirmed by FTIR (Figure 2b). Moreover, as discussed earlier, the STA molecules were supposed to be anchored to the zinc coating firmly via coordination or ionic bonding (Figure 2c).

**Figure 5:**
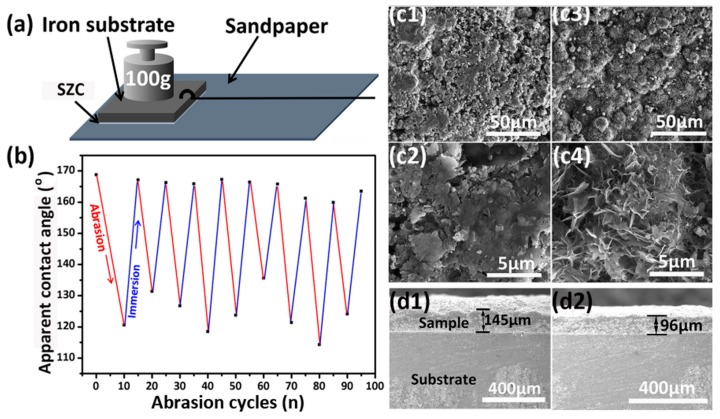
Schematic view of the setup for wearing test (**a**). Variation of apparent contact angle of the Fe-Zn-HAc-STA sample against wearing cycles (**b**). Scanning electron images for the Fe-Zn-HAc-STA sample after 10 cycles of wearing (**c1**,**c2**) and re-impregnation in acetic acid solution and ethanol solution of stearic acid (**c3**,**c4**). Scanning electron images for the cross-section of the Fe-Zn-HAc-STA sample before (**d1**) and after (**d2**) wearing for 100 cycles.

An abrading test was also performed to further evaluate the mechanical durability of the Fe-Zn-HAc-STA sample, which was subjected to a weight of 100 g while in contact with a 600 grit sandpaper at a length of 20 cm (Figure 5a). After 10 cycles, the micro/nanostructures on the Fe-Zn-HAc-STA sample were nearly destroyed (Figure 5(c1,c2)) and the apparent contact angle decreased to approximately 120°. This suggests that the outermost hydrophobic STA layer and the rough structures on the Fe-Zn-HAc-STA sample were abraded away and consequently the sample becomes hydrophobic just as the Fe-Zn sample. However, this does not mean that the coating will fail. After re-impregnation in an aqueous solution of HAc and ethanol solution of STA, the micro/nanostructures appeared on the surface (Figure 5(c3,c4)) and the coating regained the superhydrophobicity (Figure 5b). The abrading not only caused the change in the surface morphology but also decreased the thickness. As shown in Figure 5d, the thickness of the Fe-Zn-HAc-STA sample was reduced by 49 μm after 100 cycles.

### 3.3. Corrosion Resistance

**Figure 6:**
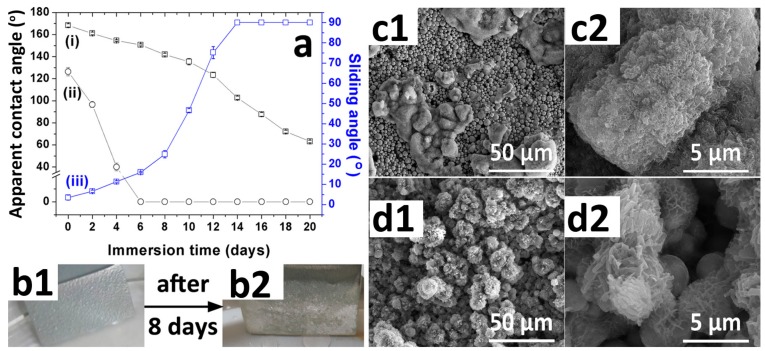
Variation of surface wettability for the Fe-Zn-HAc-STA sample (i, iii) and the Fe-Zn sample (ii) against the immersion time in the aqueous solution of NaCl (3.5 wt.%) (**a**). Digital pictures to show the underwater mirror for the Fe-Zn-HAc-STA sample just immersed into the NaCl solution (**b1**) and after 8 days (**b2**). Surface micro-morphology for the Fe-Zn sample (**c1**,**c2**) and the Fe-Zn-HAc-STA sample (**d1**,**d2**) after immersed in the aqueous solution of NaCl for 8 days.

Figure 6a shows the variation of the surface wettability for the Fe-Zn-HAc-STA sample (curves i, iii) and the Fe-Zn sample (curve ii) in the aqueous solution of NaCl for 20 days. After 6 days, the apparent contact angle of the Fe-Zn sample decreased sharply to approximately 0°; however, the Fe-Zn-HAc-STA sample still possessed good hydrophobicity (apparent contact angle of 150.7 ± 0.9° and sliding angle of 16.0 ± 0.3°). The much slower deterioration rate of hydrophobicity for the Fe-Zn-HAc-STA sample was due to the entrapped underwater air layer (EUAL) at the solid/liquid interface, which was reflected by the “silver mirror” as shown in Figure 6(b1). In other words, once the superhydrophobic Fe-Zn-HAc-STA samplewas immersed into the aqueous solution of NaCl, most of the solid surface (up to 90%) was in contact with the EUAL rather than water [25]. The EUAL blocked the diffusion of corrosive ions to the solid substrate [5] and consequently, the surface hydrophobicity of the Fe-Zn-HAc-STA sample deteriorated much more slowly as compared with the hydrophobic Fe-Zn sample. We can speculate the lasting life of the EUAL from the curves in Figure 6a. Specifically, the apparent contact angle (curve i) and the sliding angle (curve iii) for the Fe-Zn-HAc-STA sample varied very gently before 8 days and 10 days, respectively; thereafter, the variation was much faster. Moreover, the underwater “silver mirror” after 8 days as shown in Figure 6(b2) was not so obvious and became discontinuous. These data hint that the lasting life of EUAL was about 8 to 10 days. After that, statistically speaking, the STA layer rather than the EUAL was in contact with water. The surface micro-morphology of the Fe-Zn sample after immersion is shown in Figure 6c. Some area was covered by the corrosion products. In contrast, the micro/nano- hierarchical surface structures (Figure 6d) for the Fe-Zn-HAc-STA sample varied a little but were still observable, which is the morphology requirement for the superhydrophobicity.

The element content of the samples before and after immersion was measured by EDX and is summarized in Table 1. As confirmed by FTIR, XPS, and XRD earlier, the Fe-Zn sample was mainly composed of Zn MPs and epoxy resin; for the sample of Fe-Zn-HAc-STA, STA molecules were attached. Therefore, the main elements for these two samples were carbon, oxygen, and zinc. After immersion, the signal attributed to the element of chlorine emerged, which was attributed to the adsorbed NaCl or the chlorine contained corrosion products. For the Fe-Zn sample, the element content of carbon decreased sharply from 61.83 ± 6.23% to 17.48 ± 4.45%, and the oxygen increased from 6.14 ± 0.75% to 53.77 ± 7.42%. This suggests that the carbon-rich organic molecules were detached severely from the Fe-Zn sample and lots of oxygen-rich corrosion products such as Zn(OH)_2_ were formed via the following electrochemical reactions.

(1) $$\mathrm{anode}\mathrm{reaction}:\mathrm{Zn}-2e\to {\mathrm{Zn}}^{2+}$$

(2) $$\mathrm{cathode}\mathrm{reaction}:O_{2}+2H_{2}O+4e\to 4OH^{-}$$

(3) $$\mathrm{overall}\mathrm{reaction}:2Zn+O_{2}+2H_{2}O\to 2Zn{\left(OH\right)}_{2}↓$$

However, for the Fe-Zn-HAc-STA sample, the content change of carbon and oxygen was not so obvious. This is due to the barrier effect of the EUAL with a lasting life of about 8 to 10 days as discussed earlier.

**Figure 7:**
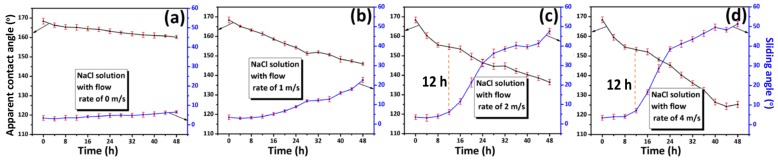
Variation of the surface wettability for the Fe-Zn-HAc-STA sample immersed in 3.5 wt.% NaCl solution for 48 h at a flow rate of 0 m/s (**a**), 1 m/s (**b**), 2 m/s (**c**) and 4 m/s (**d**).

We monitored the variation of the surface wettability in the dynamic aqueous solution of NaCl for 48 h (Figure 7). The flow rate affected the variation of the surface wettability significantly. Curves in Figure 7a (static solution) and Figure 7b (1 m/s) vary smoothly. However, as the flow rate increased to 2 or 4 m/s, similar turning points as shown in Figure 6a at 8 to 10 days appeared after a short immersion of approximately 12 h. This suggests that the lasting life of the EUAL was shortened significantly under dynamic solution with the high flow rate of 2 to 4 m/s.

**Figure 8:**
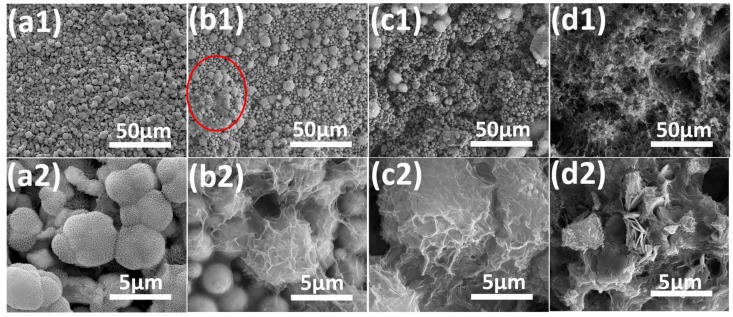
FE-SEM images for the Fe-Zn-HAc-STA samples in the aqueous solution of NaCl (3.5 wt.%) for 48 h at a flow rate of 0 m/s (**a1**,**a2**), 1 m/s (**b1**,**b2**), 2 m/s (**c1**,**c2**) and 4 m/s (**d1**,**d2**).

Figure 8 shows the micro-morphological images for the Fe-Zn-HAc-STA sample after immersed in the aqueous solution of NaCl for 48 h. The sample the underwent static corrosion still possessed a hierarchical surface microstructure (Figure 8a). As the flow rate increased to 1 or 2 m/s, some area (the circle in Figure 8(b1)) of the surface became compact and the MPs were covered by the corrosion products (Figure 8(b2,c2)). At 4 m/s, the shape of Zn MPs was difficult to recognize.

The element content of the Fe-Zn-HAc-STA sample after dynamic corrosion at different flow rates was measured by EDX (Table 2). The oxygen content increased from 8.46 ± 3.20% to 13.63 ± 3.89% at 1 m/s or to 56.47 ± 5.89% at 4 m/s after 48 h. The carbon content remained almost unchanged at 1 m/s and decreased sharply to 10.50 ± 2.14% at 4 m/s. As discussed earlier, the decreasing carbon content was due to the detachment of STA molecules and the increasing of oxygen content is due to the formation of corrosion products. These data suggest that the flow rate affects the corrosion of the superhydrophobic sample significantly. The reason is that the flowing liquid made it difficult to maintain the EUAL at the submerged superhydrophobic surface.

## 4. Conclusions

A superhydrophobic zinc coating coded as Fe-Zn-HAc-STA was prepared on the iron substrate via a facial multi-step procedure including (a) the spraying of the widely commercially available cold galvanized paint, (b) the etching by acetic acid (HAc), and (c) the hydrophobizing by stearic acid (STA). The Fe-Zn-HAc-STA sample possessed good mechanical durability and its superhydrophobicity remained after being ultrasonicated in ethanol for 100 min. After being abraded against the sandpaper for 10 cycles, the surface superhydrophobicity was lost due to the destruction of the hierarchical surface structures and the detachment of the hydrophobic STA molecules. However, after quick HAc etching and STA hydrophobizing, the sample regained the superhydrophobicity easily. The Fe-Zn-HAc-STA sample was immersed into the aqueous solution of NaCl to evaluate the corrosion resistance, and an entrapped underwater air layer (EUAL) was formed at the solid/liquid interface. The lasting life of EUAL in the static solution was 8 to 10 days and decreased sharply to 12 h as the flow rate of the corrosive solution increased to 2 or 4 m/s. We can say that the superhydrophobic surface provided extra corrosion protection of 8 to 10 days or 12 h to the zinc coating. We hope that this research may be helpful for the design of anti-corrosion superhydrophobic coatings due to the facile and fast fabrication procedure, the good mechanical durability, the easy repairability, and the good corrosion protection.

## Figures and Tables

**Table 1:** Atomic element content of the Fe-Zn sample and the Fe-Zn-HAc-STA sample before and after immersing in the aqueous solution of NaCl for 8 days.

Element	Fe-Zn		Fe-Zn-HAc-STA	
	Before Corrosion	After Corrosion	Before Corrosion	After Corrosion
C	61.83 ± 6.23%	17.48 ± 4.45%	78.07 ± 4.29%	80.63 ± 8.53%
O	6.14 ± 0.75%	53.77 ± 7.42%	8.46 ± 3.20%	7.61± 1.58%
Zn	32.03 ± 4.15%	19.58 ± 3.68%	13.47 ± 1.47%	9.24 ± 1.08%
Cl	0.00%	9.16 ± 1.51%	0.00%	2.53 ± 0.89%

**Table 2:** Atomic element content of the Fe-Zn-HAc-STA sample before and after immersion in the aqueous solution of NaCl (3.5 wt.%) for 48 h at different flow rates.

Element	Before Corrosion	After Corrosion at Different Flow Rate	
		1 m/s	4 m/s
C	78.07 ± 4.29%	80.04 ± 5.53%	10.50 ± 2.14%
O	8.46 ± 3.20%	13.63 ± 3.89%	56.47 ± 5.89%
Zn	13.47 ± 1.47%	6.17 ± 1.20%	30.82 ± 4.18%
Cl	0.00%	0.16 ± 0.02%	2.21 ± 3.55%
